# Could L2 Lexical Attrition Be Predicted in the Dimension of Valence, Arousal, and Dominance?

**DOI:** 10.3389/fpsyg.2020.552140

**Published:** 2020-12-17

**Authors:** Chuanbin Ni, Xiaobing Jin

**Affiliations:** School of Foreign Languages and Cultures, Nanjing Normal University, Nanjing, China

**Keywords:** lexical attrition, emotional words, memory, bilingualism, second language

## Abstract

The current study attended to predict L2 lexical attrition by means of a Decision Tree model (DT model) in three emotional dimensions, that is, the valence dimension, the arousal dimension, and the dominance dimension. A sample of 188 participants whose L1 was Chinese and L2 was English performed a recognition test of 500 words for measuring the L2 lexical attrition. The findings explored by the Decision Tree model indicated that L2 lexical attrition could be predicted in all the three emotional dimensions in two aspects: (1) among the three emotional dimensions, the valence dimension was the most powerful in predicting L2 lexical attrition, followed successively by the dominance dimension and the arousal dimension; (2) most of the neutral words in the three emotional dimensions were predicted to be inferior to emotional words in L2 attrition. In addition, the modified Revised Hierarchical Model for emotion could be adopted to justify the modulation of the emotion–memory effects upon L2 lexical attrition.

## Introduction

It is widely accepted that people differ in memory performance. One of the effective factors is the emotional properties of the memorized items (see Mather, 2012; Carretié, 2014; for a review). Numerous studies have shown that emotional items such as emotional words were better remembered than the neutral ones (Bradley and Lang, 1994; Ayçiçegi-Dinn and Caldwell-Harris, 2009; Pavlenko, 2012). In the case of emotional words, Bradley et al. (1992), Dolcos et al. (2005), and Weymar et al. (2015) presented the evidence that emotion improved the long-term consolidation of lexical memory, suggesting that emotion–memory effects were more likely to be seen with delayed retention. L2 attrition, which was defined as “the loss of language skills by those who have studied and then discontinued the use of L2” (Freed, 1982, p.1), is a special case for long-term memory (Ni, 2009). Thus, this study intended to explore the emotion–memory effects upon L2 lexical attrition.

### Linguistic Features Relevant to L2 Lexical Attrition

Being easily manipulated and sensitive to time, L2 words have been particularly valued by L2 attrition researchers since they began to investigate L2 attrition more than 40 years ago (see Jin and Ni, 2011; Pavlenko, 2012, for a review). The linguistic features of L2 words have been one of the core issues in L2 lexical attrition. The linguistic features were usually used in a broad sense in the field of language attrition, particularly in the field of L2 lexical attrition (Hansen, 2001; Jin and Ni, 2011; Schmid and Köpke, 2017), which might involve word class, word frequency, word length, concreteness, cognate status, polysemous senses, and so on.

Andersen (1982) was the first to analyze the linguistic features in L2 lexical attrition. He also proposed “Linguistic Feature Hypothesis (LFH),” which has been widely accepted in language attrition, to interpret the mechanisms of the relevant linguistic features in L2 lexical attrition. As reviewed by de Bot and Weltens (1991), Andersen’s LFH provided another way of looking at the attrition puzzles, with highlights on two points. One was the properties of L2 words, such as whether they were high or low in word frequency and whether they were marked or unmarked. These properties would play an important role in determining whether L2 words might be attrited or not. The other was the corresponding structures shared by L1 and L2 words. The similarity of the lexical structures between the two languages would help determine what would be vulnerable to attrition.

Guided by LFH, de Bot and Weltens (1991) analyzed the linguistic features relevant to L2 attrition at the lexical level, which involved word frequency and cognate status between L1 and L2 words. de Bot and Weltens claimed that low-frequency words would be more likely to be lost than high-frequency ones, and non-cognate L2 words, in which there was no similarity between the L1 and L2 words, would be more likely to be lost than cognate words. Subsequently, de Groot and Keijzer (2000) investigated L2 lexical attrition with a paired-associate training technique in which L2 words were paired with pseudowords. The real words were manipulated on word concreteness, cognate status, and frequency. de Groot and Keijzer found that cognates and concrete words were less susceptible to attrition than non-cognates and abstract words, while word frequency hardly affected L2 lexical attrition. With the same training technique as de Groot and Keijzer did in 2000, Tagashira (2017) explored L2 lexical attrition among Japanese EFL learners. He disclosed that concrete L2 words were less susceptible to attrition than abstract ones. However, in the investigation into the attrition of L2 nouns among adult Iranian learners of English, Marefat and Rouhshad (2007) did not find significant differences between the concrete and abstract nouns across different proficiency levels, suggesting null effect of word concreteness on L2 lexical attrition.

In addition to such possible linguistic features as word frequency, cognate status, and concreteness, Cohen (1986) compared the L2 attrition of different word classes and found that nouns were more prone to attrition than the verbs. However, Ross (2002) made a preliminary investigation into the role of word class in L2 lexical attrition and compared the relative vulnerability of nouns and verbs in the attrition of school learned French (L2). She found that nouns were better remembered than verbs, which is inconsistent with Cohen (1986). Besides verbs and nouns, Ghasemi Bagherabadi (2005, as reviewed by Marefat and Rouhshad, 2007) dealt with the attrition of the adjectives in English as L2, and Russell (2005) analyzed a particular class of function words in Japanese as L2, namely, the particles like -wa and -ga. Bagherabadi presented the evidence that L2 lexical attrition in verbs took place faster than in nouns and adjectives, while Russell showed that EL-ga and SC-ga were more easily to be attrited than -wa.

Jin and Ni (2011) covered more possible linguistic features and analyzed their roles in L2 lexical attrition with a Decision Tree model (DT model). In total, five possible linguistic features of English learned in Chinese context were examined, that is, *word frequency*, *lexical form* (in terms of the number of letters in a word), *word senses* (in the form of the number of polysemous senses), *word class*, and *concreteness*. Their results suggested that the positive linguistic features for L2 lexical attrition involved word frequency, word length, and polysemous senses and concreteness. In other words, those L2 words of low frequency and concreteness, with fewer letters and polysemous senses, were prone to L2 lexical attrition. Word class was not identified as a significant feature relating to L2 lexical attrition as reported by researchers like Ross (2002) and Marefat and Rouhshad (2007).

### Basic Components of Emotion

Although there was no absolute consensus about the basic components of emotion, the researchers shared something in common with the groups of basic components of emotion, as claimed by Arifin and Cheung (2007). In their opinion, there were two general approaches to exploring the basic components of emotion.

One was the categorical approach, by which emotion was discrete and belonged to one of a few basic categories. For example, Arifin and Cheung (2007) thought that there were five categories, *fear*, *anger*, *sadness*, *happiness*, and *disgust* and *surprise*; Smith and Ellsworth (1985) discovered six, *pleasantness*, *anticipated effort*, *certainty*, *attentional activity*, *self-other responsibility*, and *situational control*; and Bower (1981) held that there were eight, *joy*, *acceptance*, *fear*, *surprise*, *sadness*, *disgust*, *anger*, and *anticipation*.

The other was the dimensional approach. This approach did not break emotion down into a finite set but figured out a finite set of possible dimensions along which the components of emotion could be grouped. In general, emotion was often organized into a two‐ or three-dimensional structure. Since very early on in the studies on emotion, valence and arousal have been central to the dimensional classification of emotion (see Stadthagen-Gonzalez et al., 2017, for a review), which came under the general name of the two-dimensional model. In this model, valence was the most intuitive property of an emotional state and described the pleasantness vs. the unpleasantness of feelings toward an object, while arousal was defined as an energetic reaction to stimuli varying from calm to completely excited (Bakker et al., 2014; Imbir, 2016). However, more recent works including dominance as an additional dimension have concluded that the three-dimensional model was superior to the two-dimensional model (Russell and Mehrabian, 1977; Ekman and Davidson, 1995; Mehrabian, 1996; Demaree et al., 2009; Krause and North, 2016; see Bradley and Lang, 1994, for a review). The three-dimensional model (pleasure, arousal, dominance; PAD model) was first proposed by Mehrabian and Russell in 1974 (Mehrabian and Russell, 1974; see Tsai et al., 2008, for a review). In the PAD model, dominance added to the two-dimensional model represented a measure of control toward perceived feelings evoked by stimuli and varied from being under the influence of emotion to being in charge of controlling ourselves (Bakker et al., 2014; Imbir, 2016).

With regard to the order of importance for each dimension in the PAD model, researchers presented different findings with various statistical methods, like factor analysis (Osgood and Suci, 1955; Mehrabian and Russell, 1974; Russell, 1978; Bradley and Lang, 1994; Fontaine et al., 2007; Tsai et al., 2008), confirmatory factor analysis (Detandt et al., 2017) and structural equation modeling (Miniero et al., 2014). Their statistical results of the percentages accounting for the variance were presented in Table 1.

**Table 1:** Percentages accounting for the variance.

Authors	Valence	Arousal	Dominance	Total variance
Osgood and Suci (1955)	33.8	6.2	7.6	47.6
Mehrabian and Russell (1974)	24.6	23.1	12.2	59.9
Russell (1978)	44.0	30.0	11.0	85.0
Bradley and Lang (1994)	24.0	23.0	12.0	59.0
Fontaine et al. (2007)	35.3	11.4	22.8	69.5
Tsai et al. (2008)	Factor I	Factor II	Factor III	71.9
Detandt et al. (2017)	41.3	15.7	8.9	65.9

Osgood and Suci (1955) conducted factor analysis of 50 different word pairs with bipolar scales and found that 47.6% of the total variance accounted for three factors that they named evaluation (valence), activity (arousal), and potency (dominance). As indicated in Table 1, the order of importance in terms of the percentage of variance for each factor was valence (33.8%), dominance (7.6%), and arousal (6.2%). Mehrabian and Russell (1974) found similar dimensions of emotion based on the judgments of facial expressions, but in a different order: valence (24.6%), arousal (23.1%), and dominance (12.2%). The same order of importance as Mehrabian and Russell did was obtained by Russell (1978) based on the judgments of 192 adjectives denoting feelings, but the percentage of the total variance was as high as 85%, and the percentages of the three dimensions were 44.0% (valence), 30.0% (arousal), and 11.0% (dominance), respectively. Bradley and Lang (1994) employed a set of affective pictures rated with both pencil and paper and computer methods. Different rating methodologies yielded the same results based on factors analysis, that is, three factors of pleasure, arousal, and dominance accounted for 24, 23, and 12% of the variance, respectively. Fontaine et al. (2007) carried out a cross-cultural study among three language culture samples, namely, 198 Dutch-speaking samples, 188 English-speaking samples, and 145 French-speaking samples. Fontaine et al. claimed that the first dimension (valence) accounted for 35.3% of the total variance, the second dimension (dominance) for 22.8%, the third dimension (arousal) for 11.4%. They also highlighted that the overall structure could be replicated within each of the three language culture samples. Although Tsai et al. (2008) did not present the specific percentages of the total variance for each dimension, they did extract three emotional factors. Their findings indicated that the first factor was related to valence, the second to arousal, and the third to dominance. Three factors were extracted with the total variance as high as 71.90%. With confirmatory factor analysis which was different from the methods adopted by the studies mentioned above, Detandt et al. (2017) carried out an investigation with a French version of the Mehrabian and Russell’s PAD model among a population of 111 French-speaking adults. They validated the French version of the PAD model and found that the mean variance component explained by the valence dimension was 41.3% (ranging from 33.6 to 50.4), by the arousal dimension 15.7% (ranging from 8.4 to 21.3), and by the dominance dimension 8.9% (ranging from 5.9 to 11.04).

As shown in Table 1, researchers obtained contradictory results in regard to the order of importance for each dimension in the PAD model. To sum up, the valence dimension always took the first place, and the discrepancy only originated from between the arousal and dominance dimension. Most of the researchers (Mehrabian and Russell, 1974; Russell, 1978; Bradley and Lang, 1994; Tsai et al., 2008; Detandt et al., 2017) held that the arousal and dominance dimension took second and third place respectively, while only Osgood and Suci (1955) and Fontaine et al. (2007) thought that the dominance dimension was in second place and arousal in third.

### Emotion–Memory Effects

The most well-known account of how emotion could modulate memory was Freud’s theory which claimed that events injurious to the ego could be prevented from entering consciousness. Despite its importance in psychoanalytic theory, the role in determining memory has received very little experimental support (Parkin et al., 1982). In psychological experiments, the use of emotional rather than neutral stimuli was found to facilitate the rapid detection of salient events (Espuny et al., 2018). Thus, the emotional stimuli were better remembered than the neutral, which was defined as the emotion–memory effects of emotional events by Bloise and Johnson (2007). Additionally, not only salient emotional events but also learned emotional stimuli, such as words, could enhance certain cognitive processes and behavioral responses in different contexts. To be specific, as to lexical memory, the emotion–memory effects were found to be valid for both L1 (Hamann, 2001; Kensinger and Corkin, 2003; Greenberg et al., 2012; Madan et al., 2012; Ferré et al., 2013; Wierzba et al., 2018) and L2 (as reviewed by Ferré et al., 2010, 2013; Pavlenko, 2012).

#### Emotion–Memory Effects of L1 Words

In order to examine the theoretical account presented by Freud on the emotion–memory effects of L1 words, Levinger and Clark (1961) administered a word association test and a recall test with 60 words, half of which were emotional words and half neutral words (see Parkin et al., 1982, for a review). However, they carefully avoided any direct Freudian interpretation. Instead, they merely suggested that emotional responses should be subject to some form of “emotional inhibition,” the nature of which has not been clearly specified. Aiming to provide a more direct test for Levinger and Clark’s results, Parkin et al. (1982) used the same materials as Levinger and Clark did and extended the tests to a condition of delayed retention (2 min). They reported that the emotional words were significantly less remembered than the neutral words in the immediate recall tests, while the emotional words were better remembered in the delayed recall tests. With a larger sample of words than that adopted by Levinger and Clark and Parkin et al., Rubin and Friendly (1986) analyzed 925 nouns with multiple-trial free recall tests to investigate the lexical properties (orthography, imagery and meaning, word frequency, recall, emotionality, and goodness) that would make a word easy to remember. Their tests indicated that emotionality was one of the best predictors of which word was remembered.

Considering that the previous studies only took emotional words as an integrated group, Dewhurst and Parry (2000) carried out two experiments to compare the emotion–memory effects of positive, neutral, and negative words with the remember–know paradigm. Dewhurst and Parry found that the words judged to evoke a positive or negative emotional response were remembered more than emotionally neutral words. The emotion–memory effects were much stronger with negative words than with positive ones.

With the further classification teasing the positive and negative words apart, tens of studies were conducted on the emotion–memory effects of L1 words. Based on the results presented by these studies, Murphy and Isaacowitz (2008) examined the differences among positive, neutral, and negative words *via* a meta-analysis. They disclosed that emotionally valenced words (both the positively and negatively valenced) were better remembered than the neutral. Besides, some researchers compared the emotion–memory effects exclusively between the positive words and negative words as Dewhurst and Parry (2000) did, while using different experimental paradigms. For instance, with recognition tests, Greenberg et al. (2012) found that the negative words were scored much higher than the positive in the recall tests and the positive words were scored higher than the negative in the recognition tests. Moreover, both positive and negative words were scored much higher than the neutral.

The studies mentioned above were all done along only valence dimension. Actually, another dimension of emotion, the arousal dimension, has been investigated in parallel. By 1972 (see Levonian, 1972, for a review), a number of studies had measured the relation between arousal and memory, but yielding discrepant results. The key discrepancy was found to be in short-term memory, for some studies supported that higher arousal was related to better memory, while others to poorer memory. The published results for long-term memory (a few minutes) were identical, that is, higher arousal was related to better long-term memory (30 min or more). As for the interactive emotion–memory effects of valence and arousal, Kensinger and Corkin (2003) carried out six experiments with recall tests and recognition tests. They found that the relative contribution of valence and arousal was obvious to the emotion–memory effects as compared with the neutral words, and the emotion–memory effects were much stronger when words were high in both arousal and valence. However, some researchers held that arousal should work independently from valence. With the same tests as Kensinger and Corkin did, Buchanan et al. (2006) used different materials (neutral-unrelated words, school-related words, moderately arousing emotional words, and highly arousing taboo words) to address the contribution of valence and arousal to the emotion–memory effects. Their results showed that taboo words, which were both semantically related and high arousal, were remembered best. School-related words, which were high in semantic relatedness but low in arousal, were remembered better than the moderately arousing emotional words and semantically unrelated neutral words. Their results demonstrated that arousal had independent and additive effects on emotional memory. The assumption that arousal was independent and additive (see Mather, 2012, for a review) was also supported by Mather (2012), Adelman and Estes (2013), and Artur et al. (2016). They attributed the emotion–memory effects entirely to arousal, for they claimed that memory could only be enhanced for both negative and positive stimuli, provided that they were sufficiently arousing.

#### Emotion–Memory Effects of L2 Words

The first study to examine the emotion–memory effects of L2 words was carried out by Anooshian and Hertel (1994; see Ferré et al., 2010, for a review) with unexpected free recall tests in the case of late bilinguals. Although they found the emotion–memory effects of L1 words, the L2 emotional and neutral words were scored equally in unexpected free recall tests. However, different results were presented by Ayçiçegi-Dinn and Caldwell-Harris (2004) several years later. They adopted the methodology similar to that in Anooshian and Hertel (1994) but with minor modifications. For example, the materials involved five categories of words (taboo words, reprimands, positive words, negative words, and neutral words) and the tests included both recognition and recall. Ayçiçegi-Dinn and Caldwell-Harris first proved the emotion–memory effects of L2 words in both the recall and recognition tests. In order to further explore this topic, Ayçiçegi-Dinn and Caldwell-Harris (2009) conducted a study with the same set of stimuli as they did in 2004, but by different tasks at different levels of lexical processing, that is, emotional-intensity rating (a deep processing task), counting letter features (a shallow processing task), translation, and word association (additional deep processing tasks), followed by a surprise recall task. Their results claimed that emotion–memory effects were equally valid in L1 and L2 in both shallow and deep lexical processing.

As for the null emotion–memory effects of L2 words yielded by Anooshian and Hertel in 1994, the authors posited that such effects should be partially attributed to the age of L2 acquisition. Their participants were late bilinguals. The L2 words the participants acquired would not be associated as strongly with emotional experiences as the L1 words, and would consequently be less emotionally intense. In regard to the conflicting findings between Anooshian and Hertel (1994) and Ayçiçegi-Dinn and Caldwell-Harris (2004), Ayçiçegi-Dinn and Caldwell-Harris ascribed them to two possible factors. One was the language dominance. The other was the types of context relevant to the depth of lexical processing. Furthermore, Ferré and her colleagues (Ferré et al., 2010, 2013) explored the influences of the possible factors (e.g., L2 proficiency, language dominance, the type of context, the age of L2 acquisition, and the similarity between languages) upon emotion–memory effects of L2 words. Their results suggested that except for L2 proficiency, all these factors did not seem to have any effects on the memory for L2 emotional words.

### Theoretical Accounts

Various theories have been presented in regard to the emotion–memory effects in psychological studies for different purposes. Some focus on the basic components of emotion, like the PAD model (Mehrabian and Russell, 1974; Tsai et al., 2008) and the stimulus–organism–response (S–O–R) paradigm (Mehrabian and Russell, 1974; Kim et al., 2009; Lennon et al., 2011), while others focus on interpreting the cognitive processing of L2 emotional effects, like the theory of L2 disembodiment (Pavlenko, 2012), and the Revised Hierarchical Model (RHM^1^; Kroll and Stewart, 1994; Sianipar et al., 2015). Noteworthily, only the semantic-network model seeks to decipher the mechanisms of the emotion–memory effects (Glaser, 1992; De Houwer and Hermans, 1994; Kissler et al., 2006; Monnier and Syssau, 2008; Puntoni et al., 2009; see Bower, 1981, for a review).

As reviewed by Bower (1981), the semantic-network model first appeared in papers or books by Collins and Quillian (1969), Anderson and Bower (1973), Collins and Loftus (1975), and Anderson (1976). This model made a distinction between a semantic system, which was assumed to contain all the semantic knowledge, and a lexical system, which only contained linguistic knowledge (De Houwer and Hermans, 1994). In Sianipar’s view (Sianipar et al., 2015), the ability to communicate efficiently and appropriately required the vocabulary knowledge involving both the semantic/conceptual and emotional/connotative knowledge. Thus, Sianipar et al. (2015) further divided the semantic system into two subsystems, one for emotional knowledge and the other for conceptual knowledge. According to the semantic-network model, each distinct emotional dimension such as valence, and arousal, had a specific node or unit in memory that collected together many other aspects of the emotion that were connected to it by associative nodes. Some of these various linkages were innate, while others were learned and elaborated throughout acculturation. Thus, the presentation of a word (if its lexical system was activated) would activate its corresponding conceptual nodes, and at the same time the associated emotional nodes situated in the associative semantic network would also be activated (Bower, 1981; De Houwer and Hermans, 1994; Monnier and Syssau, 2008).

**Figure 1:**
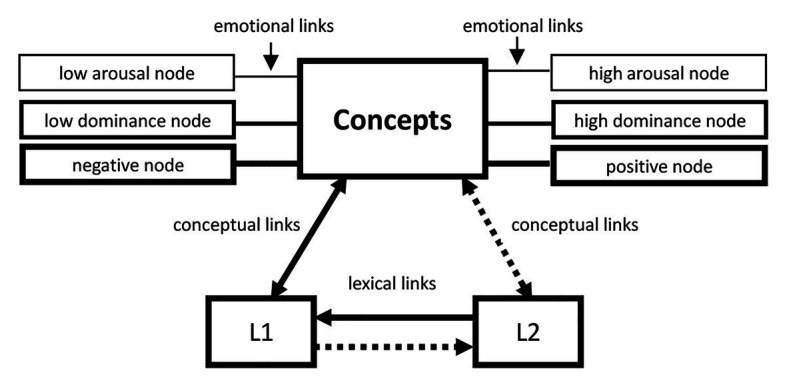
The modified Revised Hierarchical Model (RHM) for emotion.

In addition to the idea of three-in-one (lexical knowledge, conceptual and emotional knowledge) for the activation of L1 emotional words, Sianipar et al. (2015) extended the semantic-network model to interpret the emotion–memory effects of L2 words on the basis of the Revised Hierarchical Model (RHM). They assumed that a coupling of cross-language emotional and semantic processing was found to at learning-related changes in the linkage of semantic and emotional processing in L2 learning among unbalanced L2 learners. Therefore, what Sianipar et al. assumed on the emotion–memory effects could be depicted as Figure 1.^2^

As shown in Figure 1, the core of the modified RHM for emotion is the RHM. Three components of emotion, the valence, arousal, and dominance dimension, are tagged to the part of concepts. Thus, the modified RHM for emotion can be viewed on two levels. At the lower level, there is the lexical system composed of L1 and L2 words, which contains the lexical knowledge, such as the phonological representation, orthographic representation, and the pragmatic rules for using the words (Ordóñez et al., 2002). At the upper level locates the semantic system containing two subsystems responsible for the conceptual and emotional knowledge, respectively. In addition to the two original types of links in the RHM, the lexical links between L1 and L2 words, and the conceptual links between L1 or L2 words and the part of concepts, there is one new type of links in the modified RHM for emotion, the emotional links connecting the part of concepts and the three emotional components.

In light of the above discussion, we formulated the following gaps: (1) the studies on the order of importance for the three emotional dimensions yielded contradictory findings. Even worse, no study to date has ever addressed the modulation of the emotion–memory effects upon L2 lexical attrition; (2) although most of the previous studies have proved the neutral inferiority to the emotional words in valence and arousal dimension, it remains unclear whether the tendency of the neutral inferiority could also be true in the modulation of the emotion–memory effects upon L2 lexical attrition.

To fill these gaps, this study would explore the order of importance for the three emotional dimensions and examine the neutral inferiority to the emotional words. To be specific, this study would test two hypotheses:

Hypothesis 1 (order of importance): The importance of the three emotional dimensions in predicting L2 lexical attrition would be ranked in the order of the valence dimension, the arousal dimension, and the dominance dimension according to the modulation of the emotion–memory effects upon L2 lexical attrition.
Hypothesis 2 (neutral inferiority): Neutral words would be inferior to emotional words in L2 attrition in all the three emotional dimensions.

## Materials and Methods

To attest the two hypotheses presented above, we employed two types of variables, with the attrition of L2 words as the dependent variable, and the ratings of valence, arousal, and dominance for the L2 words as the independent variables (the calculation was illustrated in section Materials).

### Participants

A total of 188 participants recruited by convenience sampling were categorized into the attrited group (*n*: 151; age: 27.17, SD = 3.16; male: 96; female: 55) and the reference group (*n*: 37; age: 20.22, SD = 0.76; male: 16; female: 21), respectively. The 151 participants in the attrited group were native Chinese who had completed all systematic English learning in China, through primary school and middle school to university. They did not major in English at universities and had spent 9.33 (SD = 0.47) years learning English, and the length of English disuse was 5.63 (SD = 1.56) years (ranging from 1 to 19 years). Their exposure to English after graduation was measured by a self-perceived questionnaire with four scales (never = 0, rarely = 1, occasionally = 2, and often = 3). The self-perceived exposure to English was reported to be 0.24 (SD = 0.48), which indicated that they barely used English after graduation.

Since the lexical knowledge prior to attrition could not be feasibly obtained for the participants in the attrited group, a reference group was tested and their scores were set as an expedient baseline of the lexical knowledge prior to attrition for the attrited group. The 37 participants in the reference group were all Chinese undergraduates learning English as L2 (year of learning English: 9.35, SD = 0.44) in a Chinese university. They were at the end of the 2nd year when the recognition test in this study was carried out. The end of the 2nd year is the time when Chinese undergraduates generally stop learning English in their universities according to the National College English Teaching Syllabuses (NCETS; College English Syllabus Revision Team, 1986, 1999).

### Materials

Five hundred words for the recognition test in this study were selected among 3,894 core words by a quota sampling combined with a systematical sampling from JDEST corpus.^3^ According to their frequency presented by JDEST, 3,894 core words were divided into four groups, that is, the first, second, third, and fourth thousand words. Two-hundred words were randomly selected from the first thousand words, and then 150, 100, and 50 were obtained in sequence. In total, there were 230 nouns, 129 verbs, 108 adjectives, 12 adverbs, 7 numerals, 5 pronouns, 4 modular verbs, and 3 prepositions, and 2 conjunctions.

In the recognition test of vocabulary, each word selected had four Chinese options, only one of which was the correct translation of that word with the other three as distractors. Since the memory of the tested words of the participants was very weak, that of those stopped using English quite a long time ago in particular, the distractors were randomly assigned and were not related either in form or in meaning with the correct translation.

The participants spent about 30 min in completing the recognition test in a quiet setting with no help from the others or no reference to a dictionary or textbook. The participants in the attrited group got 375.66 (SD = 75.90) correct out of 500 words on average, while those in the reference group got 498.00 (SD = 2.79) words correct. Since the marked disproportion between male and female in the attrited (36.42% were female) and reference groups (56.76% were female) might bias the results, an independent *t* test was carried to analyze the gender difference within the attrited and reference groups respectively. No gender difference of the scores was found either in the attrited group (mean difference = 16.58; *df* = 149; *p* = 0.197; 95% CI: −41.89 to 8.72) or in the reference group (mean difference = 0.33; *df* = 35; *p* = 0.937; 95% CI: −1.57 to 2.23).

The ratings of valence, dominance, and arousal for each word tested in this study were extracted from the emotional norms of 13,915 English lemmas presented by Warriner et al. (2013). Since 31 words out of 500 words tested were absent in Warriner’s database, only 469 words were employed for analysis in this study. The properties of the tested words are shown in Table 2, and the proportions of the tested words in the three dimensions were indicated in Table 3.

**Table 2:** The properties of the tested words.

Features	Number	Mean	Std. dev	95% CI		Minimum	Maximum
				Lower	Upper		
Concreteness^a^	469	3.05	0.97	2.96	3.13	1.12	5.00
Number of letters	469	6.54	2.12	6.35	6.73	3.00	13.00
Number of syllables	469	2.08	0.96	1.99	2.17	1.00	5.00
Frequency^b^	469	2.40	0.59	2.35	2.46	0.95	4.12
Valence	469	5.54	1.23	5.43	5.65	1.68	8.21
Dominance	469	5.64	0.97	5.56	5.73	2.60	7.70
Arousal	469	4.17	0.86	4.09	4.24	2.40	6.90

a Concreteness was offered by Brysbaert et al. (2014).

b Frequency was provided by JDEST corpus, but in logarithmic values in order to eliminate data non-stationarity.

**Table 3:** The proportions of the tested words in the three dimensions.

Dimension^a^		<2	2–3	4–5	6–7	>8	Total
Valence	Number	4	49	246	168	2	469
	Percent	0.85	10.45	52.45	35.82	0.43	100%
Dominance	Number	0	28	242	199	0	469
	Percent	0	5.97	51.60	42.43	0	100%
Arousal	Number	0	211	240	18	0	469
	Percent	0	44.99	51.17	3.84	0	100%

a 9-point Likert scale was adopted.

The score of each tested word for measuring their attrition was first calculated through [(the actual correct number/the total number) × 100]. Its corresponding standardized score was then obtained through [(Z-score of the tested word) × 10 + the mean score of that word]. Finally, the measurement of L2 lexical attrition for that word was obtained by subtracting the standardized score of that word presented in the attrited group from the corresponding score of that word in the reference group.

### Data Analysis

The CHAID (Chi-square Automatic Interaction Detection) algorithm was employed as the DT model to analyze the modulation of the emotion–memory effects upon the L2 lexical attrition. The CHAID algorithm uses *Chi*-squared tests to determine which variable best predicts the outcome variable (Chan et al., 2006). The CHAID algorithm was used in this study because CHAID, in comparison to other algorithms, does not restrict the number of branches from each node to a predetermined number (Horner et al., 2010) and has no restrictions regarding the measurement level or the frequency distribution of the variables (Herschbach et al., 2004). The dependent variable was the measurement of L2 lexical attrition for 469 words, with their corresponding ratings of valence, dominance, and arousal as the independent variables. The results presented by the DT model for each level of the tree were shown in Table 4.

**Table 4:** The statistics of independent variables at each level.

Level	Variable	Independent variables statistics			Ranges estimated by decision tree
		Degree of freedom	*F*	*p*	
Level 1	Valence	df_1_ = 2; df_2_ = 1,124	14.227	0.000	≦3.90; 3.90–6.40; >6.40
Level 2	Dominance	df_1_ = 2; df_2_ = 774	7.803	0.012	≦5.00; 5.00–6.70; >6.7
Level 3	Arousal	df_1_ = 2; df_2_ = 583	7.754	0.013	≦3.20; 3.20–4.40; >4.40

As known from Table 4, *F* values of each level equaled to 14.227 (*p* = 0.000 < 0.01), 7.803 (*p* = 0.012 < 0.05) and 7.754 (*p* = 0.013 < 0.05), respectively, which indicated that this was a fairly good model.

**Figure 2:**
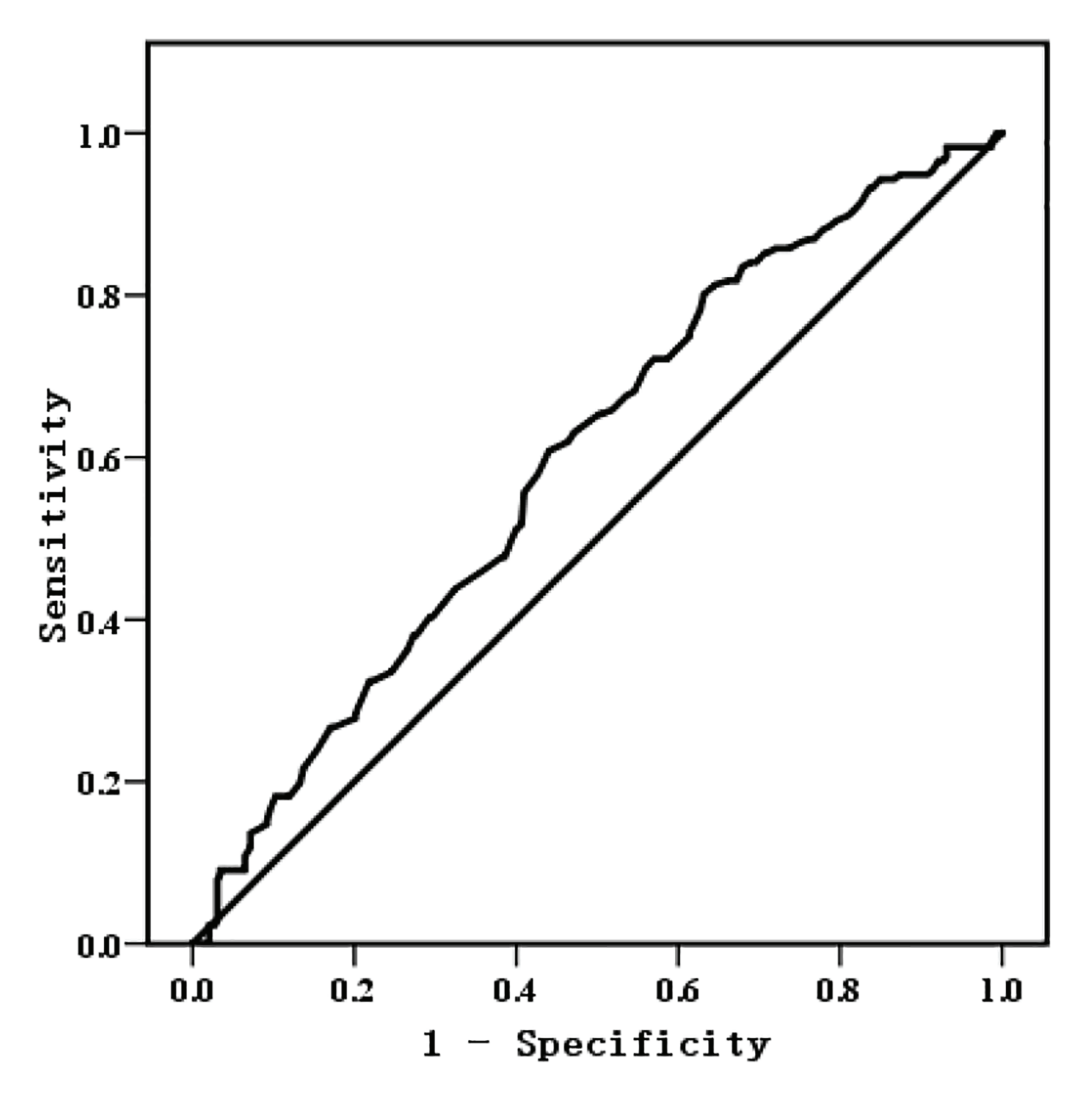
Receiver operator characteristic (ROC) curve for evaluating the Decision Tree model (DT model).

In addition to the F values of each level, we evaluated the predictive accuracy of the DT model by means of receiver operator characteristic (ROC) curve and area under the curve (AUC). The predicted values yielded by the model were assigned 1 and 0 according to whether the predicted value of a word tested was above or below the mean of the predicted values. The test variable was the measurement of L2 lexical attrition, while the state variable was the predicted values assigned. The ROC curve was plotted in Figure 2 with its test results shown in Table 5, from which we could know that AUC was 0.60 (*p* = 0.000 < 0.01, 95% CI: 0.547–0.651), suggesting that the DT model in this study could make acceptable predictions (Yang and Berdine, 2017).

**Table 5:** Test results of area under the curve (AUC).

Area	Std. error	Asymptotic sig.	Asymptotic 95% CI	
			Upper bound	Lower bound
0.60	0.027	0.000	0.547	0.651

## Results

### Levels for Predicting L2 Lexical Attrition

**Figure 3:**
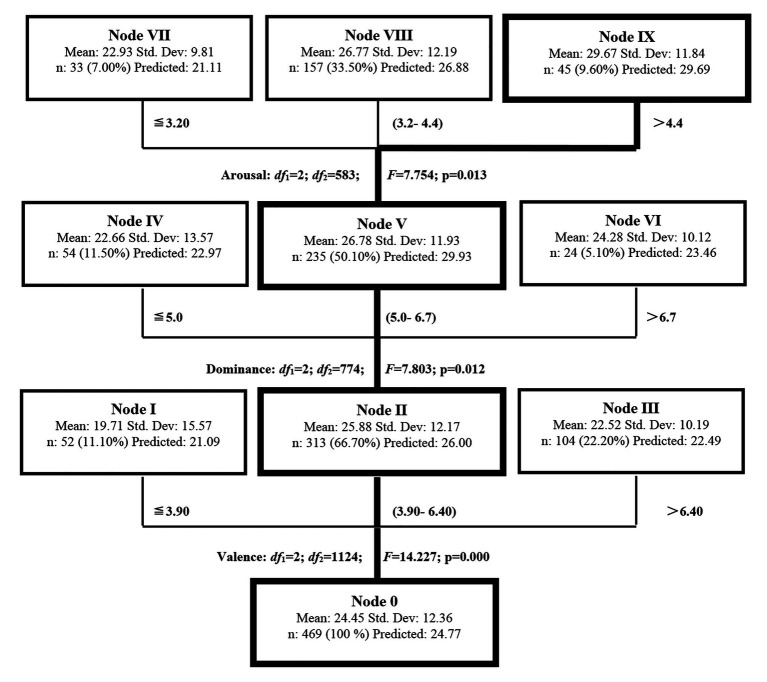
The DT model for L2 lexical attrition.

Within the model presented in Figure 3, the depth was three, and there were 10 nodes in total, 7 of which were terminal nodes, 2 internal nodes, and 1 root rode. As indicated in Figure 3 and Table 4, the decision tree grew from Root Note (Node 0) into branches at three different levels. The valence dimension was at the first level (df_1_ = 2; df_2_ = 1,124; *F* = 14.227; *p* = 0.000), the dominance dimension at the second level (df_1_ = 2; df_2_ = 774; *F* = 7.803; *p* = 0.012), and the arousal dimension at the third level (df_1_ = 2; df_2_ = 583; *F* = 7.754; *p* = 0.013). It could be seen that the most important and powerful dimension for predicting L2 lexical attrition was estimated to be the valence dimension, followed by the dominance and the arousal dimension.

As shown in Table 6 for the gain summary of each node, Node 0 (Root node) at the bottom of the decision tree involved all the 469 words tested in L2 lexical attrition, whose mean was found to be 24.45 (SD = 12.36).

**Table 6:** Gain summary of each node.

Level	Predictor	Node	Node type		N	Percent (%)	Mean	Std. dev
			By model	By attrition				
Level 0	None	0	Root node	Non key node	469	100	24.45	12.36
Level 1	Valence	I	Terminal node	Non key node	52	11.10	19.71	15.57
		**II**	**Internal node**	**Key node**	**313**	**66.70**	**25.88**	**12.17**
		III	Terminal node	Non key node	104	22.20	22.52	10.19
Level 2	Dominance	IV	Terminal node	Non key node	54	11.50	22.66	13.57
		**V**	**Internal node**	**Key node**	**235**	**50.10**	**26.78**	**11.93**
		VI	Terminal node	Non key node	24	5.10	24.28	10.12
Level 3	Arousal	VII	Terminal node	Non key node	33	7.00	22.93	9.81
		VIII	Terminal node	Non key node	157	33.50	26.77	12.19
		**IX**	**Terminal node**	**Key node**	**45**	**9.60**	**29.67**	**11.84**

Bold values indicate key node.

#### At the First Level With the Valence Dimension as the Predictor

Three child nodes, Node I (*n* = 52; 11.10%; mean = 19.71; SD = 15.57), Node II (*n* = 313; 66.70%; mean = 25.88; SD = 12.17), and Node III (*n* = 104; 22.20%; mean = 22.52; SD = 10.19), were derived from Node 0. To view these nodes horizontally, Node I and III were terminal nodes, while Node II was an internal node which was further separated into three child nodes (Node IV, V, and VI). The mean of L2 lexical attrition for Node II was 6.17 and 3.36 higher than that of Node I and III, respectively. This suggested that the words grouped in Node II were more prone to attrition than those in Node I and III, and thus Node II was the key node for predicting L2 lexical attrition on the valence dimension.

#### At the Second Level With Dominance as the Predictor

Node IV (*n* = 54; 11.5%; mean = 22.66; SD = 13.57), Node V (*n* = 235; 50.10%; mean = 26.78; SD = 11.93), and Node VI (*n* = 24; 5.10%; mean = 24.28; SD = 10.12) were extracted from Node II. Node IV and VI were terminal nodes, and Node V was an internal node, which was further segmented into three child nodes (Node VII, VIII, and IX). As for L2 lexical attrition, the mean of Node V was 4.12 and 2.50 higher than that of Node IV and VI, respectively. Thus, we could know that the words categorized in Node V were much easier to be attrited than those in the other two nodes at this level, and Node V was the key node for predicting L2 lexical attrition on the dominance dimension.

#### At the Third Level With Arousal as the Predictor

Three terminal child nodes, Node VII (*n* = 33; 7.00%; mean = 22.93; SD = 9.81), Node VIII (*n* = 157; 33.50%; mean = 26.77; SD = 12.19), and Node IX (*n* = 45; 9.60%; mean = 29.67; SD = 11.84) were partitioned from Node V. The mean of L2 lexical attrition for Node IX was 6.74 and 2.90 higher than that for Node VII and VIII. Thus, Node IX was claimed to be the key node at this level and the words grouped into this node were assumed to be attrited more than those in either Node VII or VIII.

### Ranges for Predicting L2 Lexical Attrition

Based on the statistical results shown above, we obtained three key nodes in which the groups of words were more prone to L2 lexical attrition. By defining the ranges of these groups of words rated for valence, arousal, and dominance, we would know the ranges predicted to be prone to L2 lexical attrition in each emotional dimension. Thus, the ranges estimated by the DT model for each key node and their corresponding descriptive statistics were summarized in Table 7.^4^

**Table 7:** Statistics of the three key nodes.

Level	Predictor	Key node	N	Mean	SD	Actual ranges		Ranges estimated by DT model
						Minimum	Maximum	
1	Valence	Node II	313	5.50	0.53	3.95	6.40	3.90–6.40
2	Dominance	Node V	235	5.85	0.44	5.00	6.70	5.00–6.70
3	Arousal	Node IX	45	4.84	0.37	4.50	5.90	>4.40

#### Key Node in the Valence Dimension

It is shown in Table 7 that the range estimated by the DT model went through 3.90 to 6.40, slightly different from its actual rating range (3.95–6.40) based on descriptive statistics of the words grouped in this key node. This indicated that the words whose valence rated between 3.95 and 6.40 were more prone to attrition than those whose valence ratings were either less than 3.95 or more than 6.40.

#### Key Node in the Dominance Dimension

The range estimated by the DT model was identical with the actual range, that is, from 5.00 to 6.70. This revealed that the words whose ratings of valence went between 3.95 and 6.40 and their ratings of dominance were simultaneously from 5.00 to 6.70 were easier to be attrited.

#### Key Node in the Arousal Dimension

The range estimated by the DT model was more than 4.40. It seemed that the words whose ratings in arousal went between 4.40 and 9.0 (the upmost rating in arousal) were to be more easily attrited. However, the actual range of the words for the key node on the arousal dimension went from 4.40 only to 5.90, far less than 9. Thus, we could assume that the words, which were simultaneously rated between 3.95 and 6.40 for valence, 5.00 and 6.70 for arousal, and 4.40 and 5.90 for dominance, were most sensitive to L2 lexical attrition.

**Figure 4:**
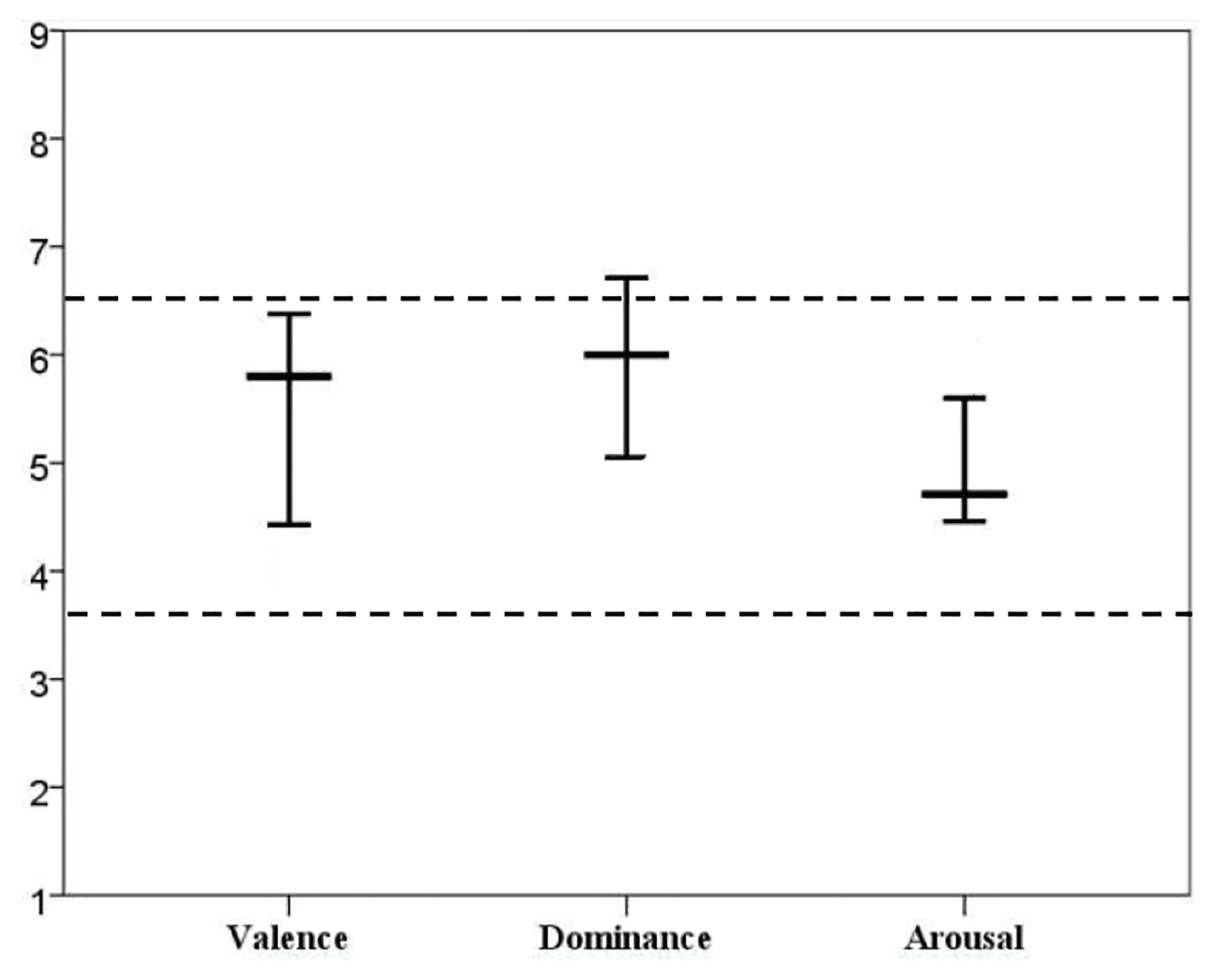
Ranges sensitive to L2 lexical attrition.

Interestingly, all the ranges predicted to be prone to L2 lexical attrition presented in this study were around 5, the middle point of the 9-point Likert scale adopted by Warriner et al. (2013). If we plotted all the three ranges predicted to be sensitive to L2 lexical attrition within a framework, we had Figure 4.

As indicated in Figure 4, the plot was divided equally into three sections by two horizontal dotted lines, which were located at 3.66 and 6.32 of the vertical axes, respectively. The three sections, less than 3.66, 3.66–6.32, and more than 6.32 were obtained by equally dividing the 9-point Likert scale. If we took the ratings of valence as an example, the words rated less than 3.66 belonged to the negative words, 3.66–6.32 to the neutral words, and more than 6.32 to the positive words. Accordingly, the words rated between 3.66 and 6.32 could be classified as the neutral words in a broad sense in both the arousal and dominance dimension. Thus, the two dotted lines in Figure 4 could be regarded as cutoff lines for defining the neutral words in valence, arousal, and dominance dimension.

As shown in Figure 4, nearly all of the ranges sensitive to lexical attrition were located in the middle area of the plot only with the upper value of the dominance dimension exceeding the boundary a little bit. Therefore, we could conclude that most of the words predicted to be sensitive to L2 lexical attrition belong to the neutral words. That is to say, the words which were either positive or negative in the valence dimension, either calm or activated in the arousal dimension, and either being in control or not in control in the dominance dimension were more resistant to L2 lexical attrition. This came to the conclusion that the neutral inferiority was predicted to be obvious in all three dimensions.

## Discussion

To our knowledge, the current study is the first to examine the modulation of the emotion–memory effects upon L2 lexical attrition in three emotional dimensions: valence, arousal, and dominance. To test two hypotheses, the current study first examined the order of importance for the three emotional dimensions in predicting L2 lexical attrition and then analyzed the modulation of emotion–memory effects on L2 lexical attrition in each dimension.

### With Respect to Hypothesis 1

Our results indicated that the valence dimension did take the first place in predicting L2 lexical attrition. The arousal and dominance dimension did not take the second and third place as assumed in Hypothesis 1, but the third and second place, respectively. As known from the studies reviewed previously in Table 1, the order of importance ranked by our results was in accordance with those obtained by Osgood and Suci (1955) and Fontaine et al. (2007) and but did not agree with those by Mehrabian and Russell (1974), Russell (1978), Bradley and Lang (1994), Tsai et al. (2008), and Detandt et al. (2017). The discrepancy only arose from the order of importance for the arousal and dominance dimension.

The underlying reasons for the discrepancy might be as follows: (1) Different statistical methods. Most of the studies reviewed in Table 1 adopted factor analysis with the exception of Detandt et al. (2017), which employed confirmatory factor analysis. As we know, factor analysis aims to identify certain unobservable factors from the observed variables by reducing the dimensionality of a set of data. It does not consider the relation between independent and dependent variables. Although confirmatory factory analysis would take the relation between independent and dependent variables into consideration, it focuses on testing whether a set of data fits a hypothesized measurement model rather than on comparing the order of importance of the independent variables in predicting a dependent variable. However, the DT model possesses some advantages over the other corresponding statistical methods employed in the previous studies. First and foremost, it can figure out the order of importance in predicting the dependent variables with the valid independent variables. In addition, the DT model can filter all the invalid independent variables so as to evaluate the importance of the valid independent variables. (2) Different retention intervals for the emotion–memory effects. Although robust and long-lasting emotion–memory effects were observed in previous studies, only three of them assessed the emotion–memory effects using longer retention intervals up to 1 year (Bradley et al., 1992; Dolcos et al., 2005; Weymar et al., 2015). In most of the studies reviewed in this study, the emotion–memory effects were assessed either immediately after the tasks (Fontaine et al., 2007; Detandt et al., 2017) or after a retention interval of a few minutes (Parkin et al., 1982), or weeks (Kalpouzos et al., 2012). However, our study covered a retention interval from 1 to 19 years. It is understandable to obtain different results since L2 lexical attrition is a special case of super-long retention interval for the lexical memory. (3) Different interactions among the three dimensions. Although valence, arousal, and dominance are independent emotional dimension, they interact with each other, particularly in lexical processing (Riegel et al., 2015). As for the interaction between the valence and arousal dimension, Yao et al. (2017) suggested that both the most negative and most positive words had higher ratings in the arousal dimension, but the increase in emotional arousal in relation to an increasing degree of negative valence seemed to be stronger than that related to an increasing degree of positive valence. For the interaction between valence and dominance, the relation between negative words and dominance was steeper than that between positive words and dominance (Fairfield et al., 2017). As far as the relation between the arousal and dominance dimension was concerned, Miniero et al. (2014) claimed that these two dimensions were more highly interacted than the relations between the other two dimensions, and they even combined the arousal and dominance dimension as a new dimension in their study.

Therefore, when we plotted the modified RHM for emotion, we employed three types of independent lines for the emotional links to illustrate the order of importance in predicting L2 lexical attrition, as shown in Figure 3. Accordingly, the lines representing the emotional links for the valence dimension were the thickest, for the dominance dimension much thinner, and the thinnest line for the arousal dimension.

### In Response to Hypothesis 2

Our results have proved that the neutral inferiority was effective in each emotional dimension. In other words, our study has extended that the neutral inferiority only proved to occur in the valence and arousal dimension to the third dimension, the dominance dimension, which has not yet been proved in previous studies. Several issues concerning the neutral inferiority are discussed as follows.

#### Asymmetric Distribution of the Three Dimensions

As shown in Table 3, although we selected the tested words on a random basis, the distribution was asymmetric in the three dimensions. There were few negative words, very few words of low dominance, and a very small amount of words of high arousal (only 18 words in total in the “>6 category”). Actually, when Dodds et al. (2015) and Iliev et al. (2016) analyzed the distribution of emotional words, they found that people would use more positive words than negative words and the words of natural human language possessed a universal positivity bias (Dodds et al., 2015).

In order to rule out the effect of asymmetric distribution that might serve as an uncontrolled variable, we ran a *post hoc* analysis with an equivalent set of words per category. Thus, 18 words were selected from each category (“<3 category,” “4–5 category,” and “>6 category”) of the three dimensions to examine the effect of L2 lexical attrition across categories.

The words selected across categories were matched in frequency and length. As to the valence dimension, the length (the number of letters) and frequency (logarithmic value) of the 18 selected words in the “<3 category,” “4–5 category,” and “>6 category” were 6.67, 6.61, 6.67, and 2.12, 2.15, 2.15, respectively. No significant differences were found across categories with one-way ANOVA either for the length (*p* = 0.996) or for the frequency (*p* = 0.981). With regard to the dominance dimension, the length of the 18 selected words in the three categories was 6.28, 6.28, 6.11, while their frequency was 2.00, 2.03, 2.12. The differences across categories were not significant in the length (*p* = 0.958) or frequency (*p* = 0.812). For the arousal dimension, the length and frequency of the three categories were 7.44, 7.39, 6.94 and 2.27, 2.20, 2.25. No significant differences were found across categories (length: *p* = 0.736; frequency: *p* = 0.919).

The L2 lexical attrition shared a similar pattern across categories in the three dimensions. The L2 lexical attrition was the highest in the “4–5 category” of the valence (33.07), dominance (28.48), and arousal (30.45) dimension, while that of both “<3 category” and “>6 category” was much lower in the valence (24.65; 24.83), dominance (21.76; 21.54), and arousal (21.22; 21.13) dimension. The results of one-way ANOVA indicated significant differences across categories in the three dimensions (valence: *p* = 0.029; dominance: *p* = 0.018; arousal: *p* = 0.015). *Post hoc* tests showed that the L2 lexical attrition of “4–5 category” was significantly higher than that of both “<3 category” (valence: MD = −8.42; *p* = 0.019; dominance: MD = −6.72, *p* = 0.015; arousal: MD = −9.23, *p* = 0.012) and “>6 category” (valence: MD = −8.24, *p* = 0.022; dominance: MD = −6.94, *p* = 0.012; arousal: MD = −9.32, *p* = 0.011), while no differences were found between “<3 category” and “>6 category” in the three dimensions (valence: MD = −0.18, *p* = 0.957; dominance: MD = 0.23, *p* = 0.936; arousal: MD = −0.09, *p* = 0.979).

Therefore, we could conclude that the data of a small set of words corroborated the results observed with the full sample.

#### The Proper Approaches to Defining the Neutral Words

In general, there are two approaches. One simple approach is to cut at the middle point of the scale for measuring emotion, as Imbir (2016) did in his study. He treated words with the given ratings of less than 5 on a 9-point Likert scale as negative in the valence dimension, and as calm in the arousal dimension, and as being in control in the dominance dimension. To Imbir, only those words with the given rating of 5 could be regarded as the neutral words in the valence and arousal dimension. The other is a classical approach that has been widely adopted in psychological studies, as in Ferré et al. (2012), Hinojosa et al. (2016), and Yao et al. (2017). For instance, Yao et al. (2017) considered words with ratings of valence ranging from 1 to 4 as negative on a 9-point Likert scale, words with the ratings of valence ranging from 4 to 6 as neutral, and words with ratings of valence ranging from 6 to 9 as positive. In our study, neither the simple approach nor the classical approach was adopted. In our opinion, the simple approach did take the neutral words into consideration, while the classical approach did not cut the scale equally. The scale for the negative and positive words ranged from 1 to 4 and 6 to 9, respectively. The scale for the negative and positive words covered three points, while the neutral words ranging from 4 to 6 only with a range of two points. Thus, we divided the 9-point Likert scale equally into three sections. Taking the valence dimension as an example, the rating less than 3.66 would be regarded as negative words, more than 6.32 as positive word, and between 3.66 and 6.32 as neutral words, respectively. In this way, we could know the range for the neutral words in all the three dimensions was between 3.66 and 6.32. As we know, in addition to figuring out the order of importance of independent variables, another one of the most distinctive advantages for the DT model over the other corresponding statistical methods is to present the ranges of each valid variable in predicting the changes of dependent variable. By comparing the ranges sensitive to L2 attrition predicted by the DT model in this study with the equally cutoff ranges (3.36–6.32) for the neutral words, we could know that almost all the words predicted to be sensitive to L2 lexical attrition belonged to neutral words.

#### Different Mechanisms From the Linguistic Features

The previous studies on the modulation of the possible linguistic features have proved that word frequency, cognate status, concreteness (Cohen, 1986; de Bot and Weltens, 1991; de Groot and Keijzer, 2000; Jin and Ni, 2011; Tagashira, 2017), and word class (Ross, 2002; Ghasemi Bagherabadi, 2005; Marefat and Rouhshad, 2007) could modulate L2 lexical attrition. In addition to these linguistic features, the emotion–memory effects could also modulate L2 lexical attrition. It seems that although both the linguistic features and the emotion–memory effects have been proved to be effective in modulating L2 lexical attrition, their underlying mechanisms might be different. According to the RHM for emotion, the linguistic features function at the lower level of the model. They are the inner features within L2 words, which could modulate the memory by only two types of links, the lexical links with L1 words, and the conceptual links with the part of concepts. However, the emotion–memory effects work at the upper level of the model. They connect with the part of concepts and will make the L2 words more salient in memory. Thus, we could claim that the mechanisms that underlay the linguistic features were different from those of emotion–memory effects in the modulation of L2 lexical attrition.

#### Different Mechanisms Underlying the Two Polarities of Each Dimension

Based on the modified RHM for emotion, we might assume that either positive or negative words in the valence dimension should be more resistant to L2 attrition, for they had more emotional links as compared with the neutral words. It was also true for the calm words or excited words in the arousal dimension and the words being in control and being not in control in the dominance dimension. However, according to this model, we could not tell whether the underlying mechanisms for the two polarities of each dimension were the same or not. In other words, we were not sure whether the links between the part of concepts and emotion for the two polarities of each emotional dimension were identical or not. In fact, this issue has been addressed by Lewis and Critchley (2003) when they reviewed the studies on emotion–memory effects. They assumed according to the semantic-network model that negative and positive words might be differentially processed. They claimed that brain regions associated with the emotional system were differentially activated by recall of information encoded in negative (left amygdala) or positive (bilateral orbitofrontal cortex) contexts. Thus, when we presented the modified RHM for emotion, we have separated the two polarities of each emotional dimension apart and each of the polarity had an independent emotional link connected to the part of concepts.

## Conclusion

To sum up, we conclude that L2 lexical attrition could be predicted in all the three emotional dimensions based on the emotion–memory effects in two aspects. First, the valence dimension was the most powerful predictor for L2 lexical attrition, followed successively by the dominance dimension and the arousal dimension. Second, most of the neutral words were inferior in L2 attrition to emotional words in all three emotional dimensions. Furthermore, the modified RHM for emotion could be adopted to justify the emotion–memory effects of L2 emotional words on L2 lexical attrition.

To our knowledge, this study is the first to explore the modulation of the emotion–memory effects upon L2 lexical attrition in three emotional dimensions, the valence, the arousal, and the dominance dimension. In addition, this study built a theoretical framework that could well justify the modulation of the emotion–memory effects. However, it is of note that this study suffered from at least two limitations. First, the L2 lexical attrition was measured on a behavioral level and the predictive accuracy may not be high enough. Thus, we must exercise caution when generalizing these findings. To raise the predictive accuracy, more sensitive neurocognitive methods such as ERPs and fMRI (Hsu et al., 2014, 2015a,b) could be employed in further studies. Second, it was really time-consuming and cognitively burdensome for a participant to consecutively finish the recognition test of 500 words. The size and representativeness of the samples, the reference group in particular, were restricted to some extent. In this case, some confronting variables such as gender differences have not been well controlled. For further studies, the words for measurement should be selected on the basis of their sensitivity to emotion–memory effects, thus more samples should be involved.

## Data Availability Statement

The raw data supporting the conclusions of this article will be made available by the authors, without undue reservation.

## Ethics Statement

The studies involving human participants were reviewed and approved by The Ethics Committee of School of Foreign Languages and Cultures of Nanjing Normal University. Written informed consent from the participants was not required to participate in this study in accordance with the national legislation and the institutional requirements.

## Author Contributions

CN is responsible for the design of the work and the analysis or interpretation of data. XJ complied the 500-word recognition test and revised critically for important content. CN and XJ both agree to be accountable for all aspects of the work. All authors contributed to the article and approved the submitted version.

### Conflict of Interest

The authors declare that the research was conducted in the absence of any commercial or financial relationships that could be construed as a potential conflict of interest.

## Footnotes

^1^Admittedly, either the BIA model (Dijkstra et al., 1999), or the BIA+ model (Dijkstra and van Heuven, 2002), or the RHM (Kroll and Stewart, 1994) might be employed in this study, for these models share the same matrix. However, only the RHM was used as the base of a theoretical framework in this study. We did so based on the following two reasons: (1) the BIA model and BIA+ model focused on the linguistic knowledge of a word, but linguistic knowledge was not our focus in this study; (2) the RHM has been used in the studies on both emotional effects and emotion–memory effects.

^2^The modified RHM for emotion presented in this study has depicted the three emotional dimensions in parallel. Actually, some researcher (Russell and Mehrabian, 1977; Bakker et al., 2014) insisted that the three emotional dimensions should be depicted in an orthogonal way. We confessed that it was a feasible way to depict the emotional dimensions in a parallel in order to save space.

^3^JDEST is a specialized corpus constructed under the supervision of Professor Yang Huizhong at Shanghai Jiao Tong University in the 1980s. It originally consisted of 1 million English words, and has been expanded to 4 million words later. The texts were sampled from different text genres in more than 30 academic domains. JDEST, since its completion, has served as a solid basis for providing information and data for the specification of the college English course in China.

^4^We listed two types of ranges, actual ranges and ranges estimated by the DT model, which might be different. The ranges estimated by the DT model were obtained based on the mathematical calculation with the DT model, while the actual ranges were defined on the basis of the minimum and maximum of the corresponding words of the key nodes. It is worth noting that the corresponding words of the key nodes have already been selected at least once or twice by the DT model and their ranges might be a little bit narrower than the ranges estimated by the DT model.
